# Conjugation to the Cell-Penetrating Peptide TAT Potentiates the Photodynamic Effect of Carboxytetramethylrhodamine

**DOI:** 10.1371/journal.pone.0017732

**Published:** 2011-03-14

**Authors:** Divyamani Srinivasan, Nandhini Muthukrishnan, Gregory A. Johnson, Alfredo Erazo-Oliveras, Jongdoo Lim, Eric E. Simanek, Jean-Philippe Pellois

**Affiliations:** 1 Department of Biochemistry and Biophysics, Texas A&M University, College Station, Texas, United States of America; 2 Department of Chemistry, Texas A&M University, College Station, Texas, United States of America; City of Hope National Medical Center and Beckman Research Institute, United States of America

## Abstract

**Background:**

Cell-penetrating peptides (CPPs) can transport macromolecular cargos into live cells. However, the cellular delivery efficiency of these reagents is often suboptimal because CPP-cargo conjugates typically remain trapped inside endosomes. Interestingly, irradiation of fluorescently labeled CPPs with light increases the release of the peptide and its cargos into the cytosol. However, the mechanism of this phenomenon is not clear. Here we investigate the molecular basis of the photo-induced endosomolytic activity of the prototypical CPPs TAT labeled to the fluorophore 5(6)-carboxytetramethylrhodamine (TMR).

**Methodology/Principal Findings:**

We report that TMR-TAT acts as a photosensitizer that can destroy membranes. TMR-TAT escapes from endosomes after exposure to moderate light doses. However, this is also accompanied by loss of plasma membrane integrity, membrane blebbing, and cell-death. In addition, the peptide causes the destruction of cells when applied extracellularly and also triggers the photohemolysis of red blood cells. These photolytic and photocytotoxic effects were inhibited by hydrophobic singlet oxygen quenchers but not by hydrophilic quenchers.

**Conclusions/Significance:**

Together, these results suggest that TAT can convert an innocuous fluorophore such as TMR into a potent photolytic agent. This effect involves the targeting of the fluorophore to cellular membranes and the production of singlet oxygen within the hydrophobic environment of the membranes. Our findings may be relevant for the design of reagents with photo-induced endosomolytic activity. The photocytotoxicity exhibited by TMR-TAT also suggests that CPP-chromophore conjugates could aid the development of novel Photodynamic Therapy agents.

## Introduction

Cell-penetrating peptides (CPPs) are used to deliver molecular cargos into live cells [1]. Cells internalize CPP-cargo conjugates through efficient endocytic mechanisms. This material, however, often remains trapped inside endosomes and the delivery of the cargo into the cytosolic space of cells is inefficient. To increase the delivery efficiency of CPPs, the endosomolytic activity of these reagents needs to be improved. When labeled with fluorescein, Alexa fluors or cyanine fluorophores, arginine-rich CPPs could be released from endosomes by irradiation with lasers for extended periods of time [2], [3], [4]. Photo-induced endosomal release of CPP conjugates has been used to increase the delivery of proteins such as p53 into live cells [3]. This light-based strategy has also been used to photo-induce the cytosolic delivery of RNA molecules and to achieve spatial and temporal control of gene expression by RNA interference [4]. The observed photo-induced endosomal release is thought to involve the formation of reactive oxygen species (ROS) generated from excitation of the fluorophore and the subsequent disruption of endosomal membranes by these species. This, however, has not been demonstrated. Furthermore, the precise mechanisms involved in this process have not been investigated.

Here we examined the photolytic activity of TAT labeled with the fluorophore 5(6)-carboxytetramethylrhodamine (TMR). We show that TMR-TAT causes rapid and efficient endosomal membrane disruption in the presence of light. Remarkably, however, this is accompanied by the rapid destruction of cells. These effects involve the targeting of TMR by TAT to cellular membranes and the formation of singlet oxygen within membranes. Interestingly, TMR itself has a low singlet oxygen quantum yield and displays no photolytic activity and no photocytotoxicity. Therefore, our data indicate that TAT can convert an innocuous chromophore into a potent photolytic agent.

## Methods

### Peptide design and synthesis

To test the photoendosomolytic activity of CPP-fluorophore conjugates, four peptides conjugated to the fluorophore 5(6)-carboxytetramethylrhodamine (TMR) were examined: the CPP TAT (GRKKRRQRRR), retro-inverso TAT (riTAT, rrrqrrkkrgy), the CPP R9 (RRRRRRRRR), and K9 (KKKKKKKKK). TMR was chosen as a model fluorophore because it is not toxic to cells in both the absence and presence of light. It is also commonly used for live cell imaging and it is synthetically readily accessible. Finally, the fluorescence of TMR is not as pH-dependent as that of fluorescein. Therefore, TMR is not affected by the acidic pH of endosomes to the extent that fluorescein is. Peptides were synthesized by solid-phase peptide synthesis using standard F-moc chemistry and purified by HPLC. In TMR-TAT and TMR-R9, the amino terminus of the peptide was directly coupled to TMR. riTAT, a peptide in which the TAT sequence is reversed and the constituent amino acids have a D rather than a L-configuration, was prepared to obtain an analogue of TAT that would be resistant to degradation by cellular proteases. TMR was introduced at the N-terminus of the peptide. TMR-K9 is comprised of 9 positively charged lysine residues and serves as a control. This peptide is endocytosed by cells and therefore it is localized inside endosomes like TAT or R9. However, K9 does not have the cell penetration activity attributed to TAT and R9.

TMR-TAT (TMR-GRKKRRQRRRG-NH_2_) expected mass: 1865.0 Da, observed mass: 1866.1 Da. TMR-R9 (TMR-RRRRRRRRRG-NH_2_) expected mass: 1893.2 Da, observed mass: 1894.4 Da. TMR-riTAT (TMR-rrrqrrkkrgy-OH) expected mass: 1973.3 Da, observed mass: 1975.3 Da. TMR-K9 (TMR-KKKKKKKKK-NH_2_) expected mass: 1583.0 Da, observed mass: 1583.3 Da.

### Cell-based assays

HeLa (human cervical adenocarcinoma), COS-7 (SV40 transformed African green monkey kidney fibroblast-like cell line) were obtained from ATCC. COLO 316 (human ovarian carcinoma) were obtained from Robert Burghardt (Department of Veterinary Integrative Biosciences, Texas A&M University) [5]. Cells were cultured in DMEM supplemented with 10% fetal bovine serum (FBS) and maintained at 37°C in a humidified environment with 5% CO_2_. Cells were plated in 8-well dishes so that the cells were 70% confluent after 24 h, washed 3 times with L-15 media, incubated with 3 to 10 µM peptides at 37°C for 1 h, washed 3 times with L-15, and imaged. Alternatively, cells were incubated in L-15 at 4°C to inhibit the endocytic uptake of peptides. In this case, cells were imaged with the peptide still present in solution and the low temperature was maintained for the entire duration of imaging. For photosensitization, cells were exposed to light (λ_ex_ = 560±20 nm) for the indicated time points. Plasma membrane disruption was detected by addition of the cell-impermeable DNA stain SYTOX Blue (5 µM) during or after photosensitization.

Whole blood was purchased from Gulf Coast Regional Blood Center (Houston, TX). Erythrocytes were centrifuged for 5 min at 1500 g and the erythrocyte pellet was resuspended with PBS. This was repeated three times to remove plasma and buffy coat. The erythrocytes (50% volume in PBS) were diluted in PBS to a final concentration of 0.25%. Indicated concentration of peptide was added to the medium and the samples were added to the wells of an 8-well chamber glass slide (Nunc). Cells were typically allowed to settle to the bottom of the dish for 5 minutes prior to imaging so as to obtain a layer of cells in the focal plane.

### Imaging

Cells were placed on an inverted epifluorescence microscope (Model IX81, Olympus, Center Valley, PA) equipped with a heating stage maintained at 37°C. The microscope is configured with a spinning disk unit to perform both confocal and wide-field fluorescence microscopy. Images were collected using a Rolera-MGI Plus back-illuminated EMCCD camera (Qimaging, Surrey, BC, Canada). Images were acquired using bright field imaging and two standard fluorescence filter sets: Texas Red (Ex = 560±20 nm/Em = 630±35 nm), and CFP (Ex = 436±10 nm/Em = 480±20 nm). The excitation light was from a 100 W halogen lamp (Olympus USH 1030C) passed through the filter cubes and 40 or 100× objective lenses. Neutral density filters (ND 1, 2, 3, or 4 on the instrument, corresponding to 100, 25, 12.5, or 5% transmittance) and different exposure times were used to modulate the amount of light samples were exposed to. The bright field and fluorescence intensities of cells and ghosts were measured using the SlideBook 4.2 software (Olympus, Center Valley, PA). To determine the percentage of cells stained by SYTOX® Blue, cells were imaged with a 20× objective by phase contrast. Ten to twenty images were acquired for each experiment. The total number of cells in a given image was determined from the phase contrast image while the number of dead cells was determined by identifying cells containing a blue fluorescent nucleus stained by SYTOX® Blue. Cell viability was determined by establishing a ratio of dead cells/total number of cells for each sample (at least 1000 cells were counted in each experiments and each experiments were repeated 3 times).

Irradiances at the specimen were 100 mW/cm^2^ when no neutral density filter and no objective were present in the light path (and, for instance 5 mW/cm^2^ when ND4 was inserted). Irradiances were measured using a monochromic photometer (model 840-c, Newport, Irvine, CA). Irradiation area has a diameter of 1.3 cm without objective but the light beam is focused into an area of 3×10^−3^ cm^2^ by the 100× objective. Irradiances can therefore be approximated to be at 21 or 420 W/cm^2^ with ND4 or ND1, respectively. Irradiances provided in the figure legends are based on these calculations. To confirm that the irradiances measured on the microscope were accurate, experiments were also reproduced, when possible, using light illumination with a collimated light source from Oriel (Stratford, CT) equipped with a 500 W Hg lamp. Selective irradiation at 560 nm was performed using an analytical line filter (Oriel, 9.4 nm bandwidth). In this case, irradiances of the light beam (diameter of 2.5 cm) could be measured more precisely using the monochromic photometer.

## Results

### Light-exposure of TMR-TAT contained inside endosomes causes endosomal release and cell death

**Figure 1:**
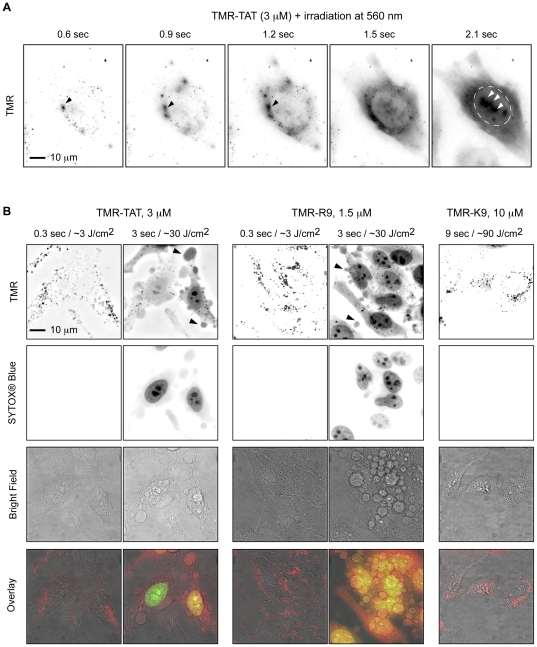
Photolytic effects of TMR-TAT after endocytosis in HeLa cells. A) Light irradiation causes escape of TMR-TAT from endocytic organelles into the cytosol. Hela cells were incubated with TMR-TAT (3 µM), washed, and irradiated at 560±20 nm through a 100× objective on a wide-field microscope. Images were acquired in a time-lapse experiment with a light excitation of 300 ms and an interval of 2 seconds. The time displayed on the images represents the total light exposure time. The TMR fluorescence signal is represented as inverted monochrome (black = fluorescent signal, white = no signal). The TMR signal, initially in a punctate distribution, can be seen to diffuse away from individual endocytic organelles upon irradiation (black arrows). The perimeter of the nucleus is highlighted by a dashed line in the last image and the signal from TMR-TAT presumably accumulated at nucleoli is indicated with white arrows. B) Photosensitization of TMR-TAT or TMR-R9 endocytosed by cells causes plasma membrane damage and cell death. In contrast, cells containing TMR-K9 remain viable and the punctate distribution of TMR-K9 is not affected by the light irradiation under the conditions tested. The fluorescence signals of TMR and SYTOX® Blue are represented as inverted monochrome or pseudo-colored red and green in the overlay image, respectively.

**Figure 2:**
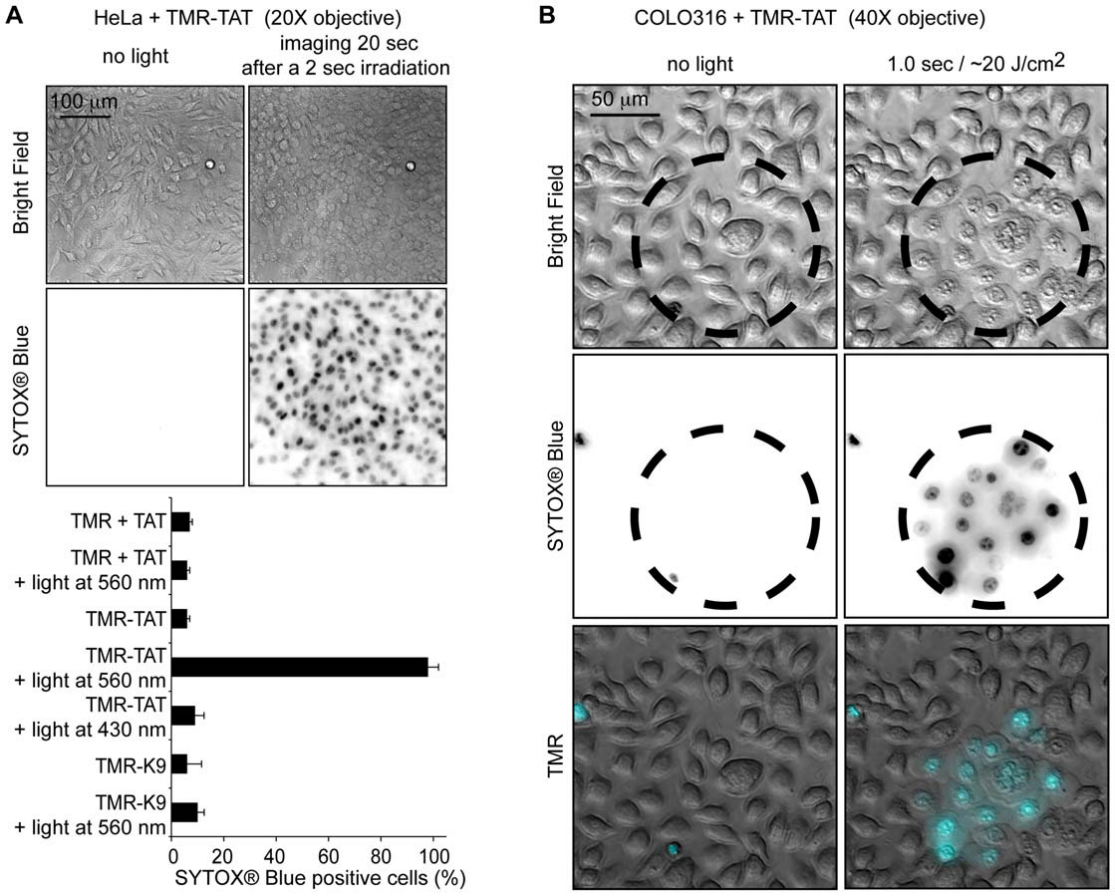
The photolytic and photocytotoxic effects of TMR-TAT occur rapidly and in all irradiated cells. A) Irradiation of TMR-TAT endocytosed by cells causes plasma membrane blebbing and permeabilization (SYTOX® Blue staining) within 20 seconds after 2 sec irradiation at 560 nm(the radiant exposure is approximately 40 J/cm^2^). Cell destruction is observed in more than 95% of the cells irradiated at 560 nm. In contrast, no photocytoxicity is observed when cells treated with TMR-K9 (10 µM) or TMR (3 µM) and TAT (3 µM) are irradiated under similar conditions. Cells incubated with TMR-TAT but irradiated at 430 nm are not destroyed (excitation wavelength of SYTOX® Blue but not of TMR, radiant exposure was also approximately 40 J/cm^2^). The histogram represents the average percentage of cells stained by SYTOX® Blue after peptide and light treatment (the number of cells examined is at least 3000) and the error bars represent the standard deviation (experiments were reproduced at least 3 times). B) The cytotoxic effect of TMR-TAT is limited to only irradiated areas. COLO 316 cells were incubated with TMR-TAT (3 µM) for 1 h. The cells were washed with fresh media and endocytosis of TMR-TAT was confirmed by fluorescence microscopy. The cells within the circled area were exposed to light at 560 nm. Morphological changes and SYTOX® Blue staining (inverted monochrome or pseudo-colored cyan) are only observable in the irradiated area.

The effect of light irradiation on cells that have endocytosed TMR-TAT was first investigated. HeLa cells were incubated in the presence of TMR-TAT (3 µM) for 1 hour at 37°C. After washing the cells, the sample was placed on a microscope and the cellular distribution of the peptide was assessed by fluorescence imaging. The peptide initially showed a punctate intracellular distribution consistent with their accumulation within endocytic organelles (TAT typically requires higher concentrations than the one used here to penetrate the cytosolic space of cells directly) (Fig. 1) [6]. Fluorescence excitation of the cells on the microscope, however, rapidly caused the release of the fluorescent peptides from endosomes into the cytosol. As shown in Figure 1A, the escape from individual endosomes was observed after only a few hundred milliseconds of light exposure at 560 nm. Within seconds, the content of many endosomes was released into the cell and the peptides were distributed throughout the cell. The peptide also accumulated at the nucleoli, a known effect of TAT [7]. Significantly, photo-induced endosomal release was accompanied by dramatic blebbing of the plasma membrane and cell shrinkage (Fig. 1B). The irradiated cells also became permeable to the cell-impermeant dye SYTOX® Blue, indicating that their plasma membranes were compromised and that the cells underwent rapid necrosis (Fig. 1B shows the morphological changes cells undergo after irradiation in details while Fig. 2A illustrates that this phenomenon occurs for all irradiated cells). The viability of non-irradiated cells was, in contrast, not affected by TMR-TAT (Fig. 2B); we found the cytotoxicity of the peptide to be negligible in the absence of light at concentrations up to 25 µM (supporting information Fig. S1).

TMR-R9 and TMR-riTAT had similar photoendosomolytic and photocytotoxic activities as TMR-TAT, indicating that an arginine-rich composition might be sufficient for these activities (Fig. 1B and S2). Like TMR-TAT, TMR-K9 was endocytosed by cells (higher concentration was used to get equal or greater amount of material inside endosomes, Fig. 1B and S3). However, irradiation of cells containing TMR-K9 caused no endosomal release or cell death under the same conditions of irradiation as TMR-TAT (Fig. 1A). TMR alone was not endocytosed to a detectable level and irradiation post-incubation did not cause cell death (data not shown). Together these data suggest that the presence of CPPs like TAT or R9 is important for the observed photo-induced endosomal lysis and cell-death. In addition, these activities cannot be solely attributed to the endocytosis of the TMR-CPP conjugates as TMR-K9 did not have any observable effects despite similar endocytic uptake. No endosomal release and no cell death was observed when TMR-TAT-treated cells were exposed to light of wavelengths not absorbed by TMR (e.g. 436 or 470 nm). Moreover, irradiation of cells incubated with unlabeled TAT and irradiated at the TMR excitation wavelength did not die (Fig. 2A). This suggests that excitation of the fluorophore is required to achieve photosensitization and that TAT and light alone do not induce endosomal release or cell death (Fig. 2A). Irradiation of cells co-incubated with both TAT and TMR did not result in cell death, suggesting that conjugation of TMR to TAT is required for this effect. Finally, the photo-induced activities of TMR-TAT could be reproduced in other cell lines, such as COLO 316 and COS-7, indicating that the effects observed are not specific to HeLa cells (Fig. 2B and 4).

### Endocytic degradation of TAT abolishes photosensitization

**Figure 3:**
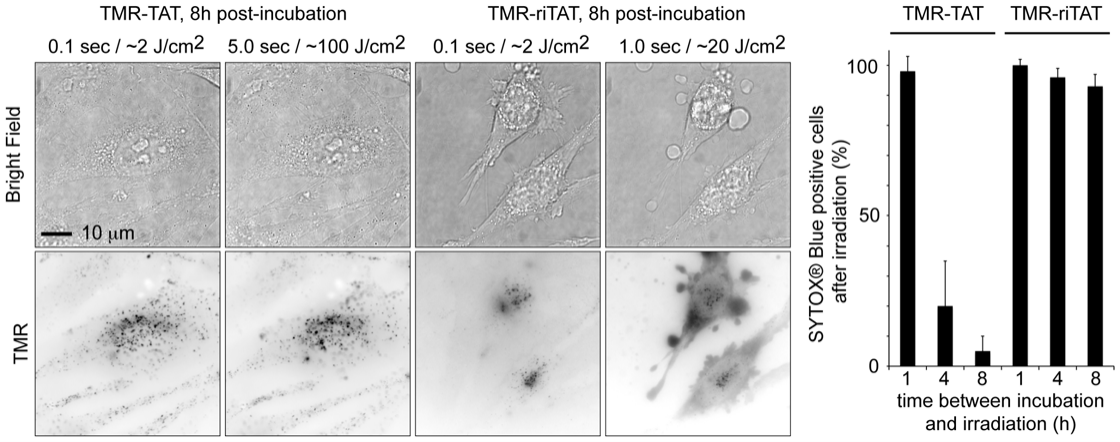
Degradation of TMR-TAT in endocytic organelles abolishes photo-induced endosomal release and photocytotoxicity. HeLa cells were incubated with TMR-TAT or TMR-riTAT (3 µM) for 1 h, washed, and incubated at 37°C for an additional 1, 4 or 8 hours. Irradiation of cells incubated with TMR-riTAT led to the cytosolic distribution of the TMR fluorescence signal, plasma membrane blebbing (as seen in the bright field image) and permeabilization (represented in histogram) under all tested conditions. In contrast, these photo-induced effects are dramatically reduced for TMR-TAT as the time between peptide incubation and irradiation is increased. The histogram represents the average percentage of cells stained by SYTOX® Blue after peptide and light treatment (the number of cells examined is at least 3000) and the error bars represent the standard deviation (experiments were reproduced at least 3 times).

The previous experiments suggest that TMR and TAT have to be conjugated to one another to achieve the light-induced endosomal release and the observed cell-death (Fig. 2A). To confirm these results, the activities of TMR-TAT and TMR-riTAT were compared as a function of the time spent within the endocytic pathway. One would expect that TMR-TAT would accumulate within late endosomes and lysosomes over time and that the TAT moiety might be degraded by proteases present in these organelles. If TAT is required for the lytic activity of TMR-TAT, degradation of the peptide should therefore abolish the activity of the compound. On the other hand, riTAT should be resistant to proteolysis and its lytic activity should not be dependent on the incubation time. HeLa cells were treated with TMR-TAT or TMR-riTAT for 1 hour to allow for endocytosis. The cells were then washed and incubated for an additional 1, 4, or 8 hours in L-15 media to allow for the accumulation of the endocytosed peptides into late endosomes and lysosomes. The cells were then observed under the microscope and treated with light as described in the previous paragraph. As expected, incubation time did not appear to have an effect on the activity of TMR-riTAT as both rapid endosomal release and cell-death were observed under all conditions tested (Fig. 3). In contrast, while endosomal release and cell-death were observed when cells treated with TMR-TAT were incubated for 1 hour, these effects were reduced or completely abolished at the 4 and 8 h time points, respectively (Fig. 3). Together, these results indicate that it is TMR-TAT that participates in the activities observed and not molecular species that might be obtained by degradation of the peptide within the endocytic pathway.

### TMR-TAT photosensitization causes plasma membrane damage and cell lysis

**Figure 4:**
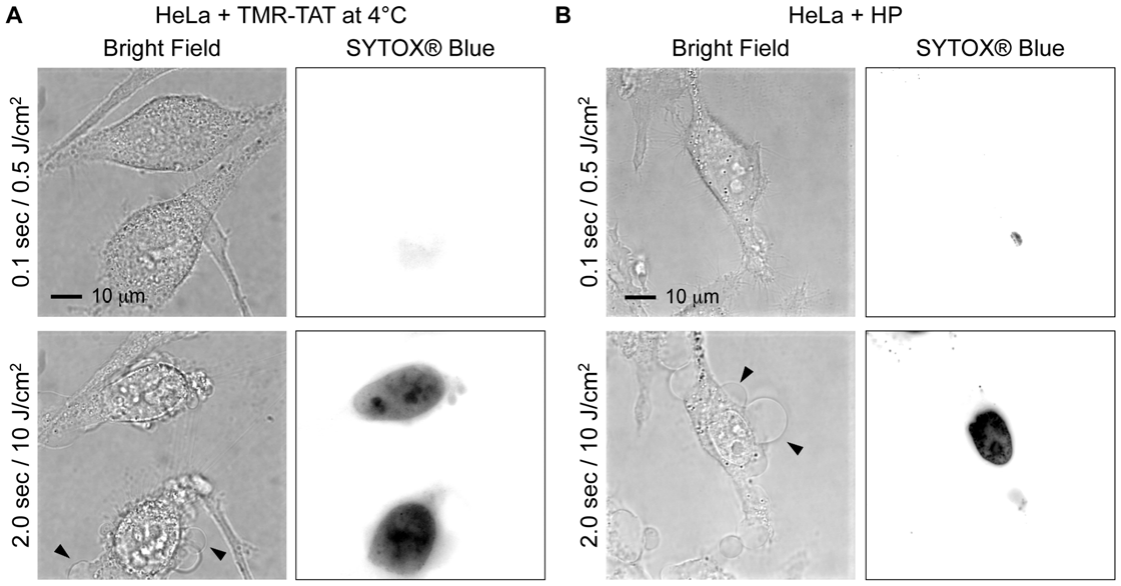
Photosensitization of extracellular TMR-TAT causes plasma membrane blebbing, plasma membrane permeabilization, and cell shrinkage. A) HeLa cells were incubated with TMR-TAT (3 µM) at 4°C to block endocytosis of the peptide. Images represent the bright field image and the fluorescence image of SYTOX® Blue (inverted monochrome). B) The effects of photosensitization of hematoporphyrin (HP) on cell morphology are similar to those obtained by photosensitization of TMR-TAT. HP was incubated with HeLa cells for 1 hour, washed with fresh L-15 media, and irradiated at 560 nm. Black arrows in A) and B) highlight the membrane blebbing observed during light exposure.

The disruption of lysosomes by photosensitizers or lysosomotropic compounds has been shown to induce cell-death [8], [9], [10]. It is therefore possible that the endosomal release observed in the experiments of Figure 1 might be necessary to cause cell death. To investigate whether this is the case, we examined if light-induced cell-death could be observed when the peptides were only localized at the plasma membrane of cells. For these experiments, HeLa cells were first incubated with the peptides (3 µM) at 4°C to block endocytosis. Cells were then irradiated with the peptides still present in the media (the peptide is not retained at the plasma membrane when cells are washed). As shown in Figure 4, cells incubated with TMR-TAT underwent dramatic cytoplasmic extrusion, membrane blebbing, shrinkage and cell death (SYTOX® Blue staining) upon light irradiation. Cells were then washed and observed by fluorescence imaging. Live cells in areas that were not irradiated did not contain fluorescent endosomes, demonstrating that the cells in this assay did not internalize the peptides. Cells incubated with either TMR-K9 or TMR alone at similar concentrations (determined by TMR absorbance) did not die under the same conditions of irradiation. On the other hand, the membrane damage and cell death obtained after TMR-TAT photosensitization were also observed when hematoporphyrin (HP) or hematoporphyrin derivative (HPD) were used (Fig. 4B). HP (or porphyrin impurities present in commercially available HP) and hematoprophyrin derivative are relatively hydrophobic photosensitizers that cause membrane damage and cell killing upon light irradiation [11]. Considering that singlet oxygen is expected to cause damage in close proximity to where the photosensitizer is located, accumulation of HP and HPD into membranes is believed to be critical to their photodynamic action on membranes. The similar destruction of membranes observed between HP and TMR-TAT suggests that TAT positions TMR in close proximity to membranes. In contrast, TAT-K9 would fail to induce a significant photocytotoxic effect because it doesn't localize at the membranes in the same manner. Because TMR-TAT is excluded from the intracellular space in these experiments, these results also suggest that photosensitization of TMR-TAT at the plasma membrane is sufficient to induce membrane blebbing, SYTOX® Blue permeation, and cell death. In other words, endocytic uptake of TMR-TAT by cells is not required to achieve light-induced cell-death as long as the peptide is localized at the plasma membrane.

**Figure 5:**
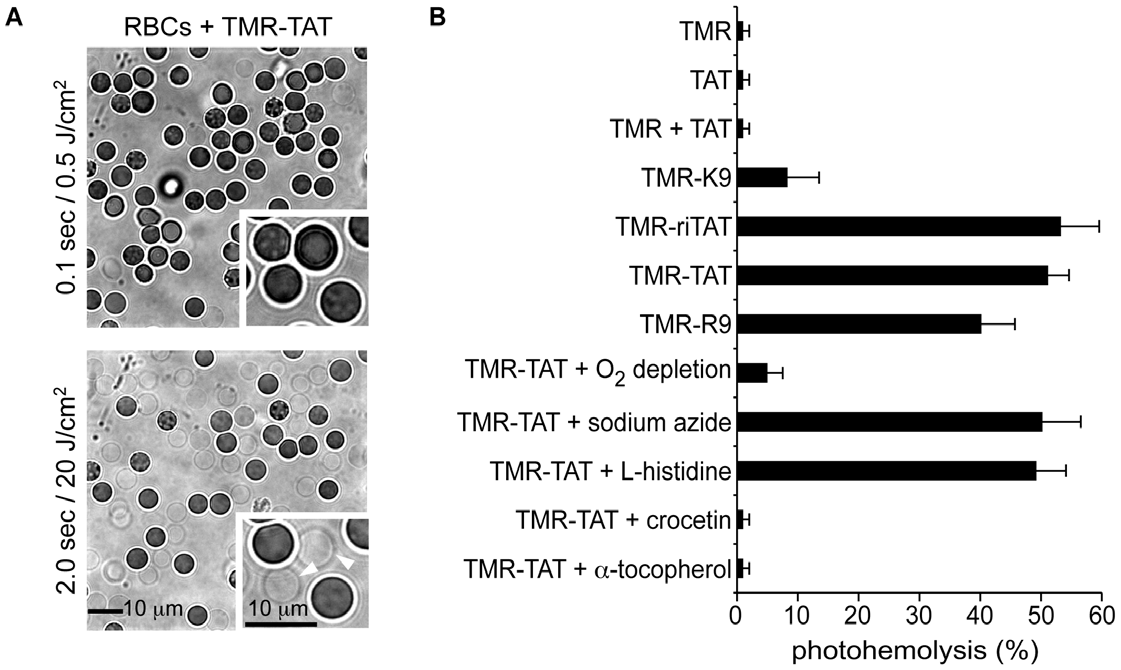
Photosensitization of TMR-TAT causes the lysis of red blood cells by the production of singlet oxygen in their membrane. A) RBCs incubated with TMR-TAT (2 µM) have either a concave or crenated morphology initially. Irradiation of the sample at 560 nm causes formation of spherical cell ghosts (highlighted with white arrows in inserts) that lose their contrast in bright field images as the cells lyse. B) Percentage of lysed RBCs as a function of the compounds present in the media. TMR and TMR containing peptides were used at 2 µM. All experiments were performed by irradiating the samples at 560 nm for 400 msec (∼8 J/cm^2^). The data in the histogram represents the average of 4 experiments and the error bars correspond to the standard deviation. No lysis was observed for any of the samples in the absence of light.

To further confirm that the fluorescent peptides are acting on membranes when irradiated, the effect of TMR-TAT, TMR-R9, and TMR-riTAT on the membrane of red blood cells (RBCs) was examined. RBCs do not typically endocytose extracellular material to significant levels [12], [13]. As a result, the effects of the peptides on the plasma membrane can be examined specifically and quantitatively. Damage to the membrane of RBCs and cell lysis can be easily monitored and quantified by bright field microscopy [14]. RBCs were incubated with TMR-TAT and TMR-riTAT (1 to 5 µM, control experiments indicated that TMR-TAT and TMR-riTAT do not penetrate RBCs under these conditions, Fig. S5). No lysis was observed in the absence of irradiation of TMR (white light was used for bright field imaging, but this has no effect on the cells). Many RBCs, however, adopted a crenated morphology upon incubation with the peptides (Fig. 5A). Crenation is indicative of a disturbance in the RBCs' membrane, an effect often observed for amphiphilic compounds, suggesting that the peptide might partially partition in the lipid bilayer [15], [16]. When observed by fluorescence imaging (at low light intensity to avoid photosensitization of TMR), the peptide's fluorescence was diffusely distributed in the media (Fig. S5). Together, these results indicate that the fraction of peptide that might interact with the RBC membranes is small in comparison to the fraction of peptide which partitions in solution. RBCs that had been unaffected by incubation with TMR-TAT for a prolonged period of time in the absence of light (10 min) started lysing immediately upon excitation with light (Fig. 5A). Formation of cell ghosts could be clearly identified by bright field imaging as RBCs leaked and lost their optical contrast (Fig. 5A). The number of lysed ghosts was proportional to the radiant exposure (Fig. S5). The ghosts formed initially had a spherical shape with a diameter approximately equal to that of non-irradiated spherical and intact RBCs. Extended exposure to light however caused a dramatic shrinking of the membrane, suggesting that damage to the lipid bilayer might continue well after lysis has occurred (Fig. S5). Consistent with the results observed with HeLa cells, TMR alone had no lytic effect on RBCs even after prolonged light exposure (Fig. 5B). TMR-K9 was 5-fold less efficient than TMR-TAT and TMR-riTAT in causing photohemolysis, suggesting again that some key features of TAT contribute to the potentiation of TMR photosensitization.

### The mechanism of cell death involves singlet oxygen generation

**Figure 6:**
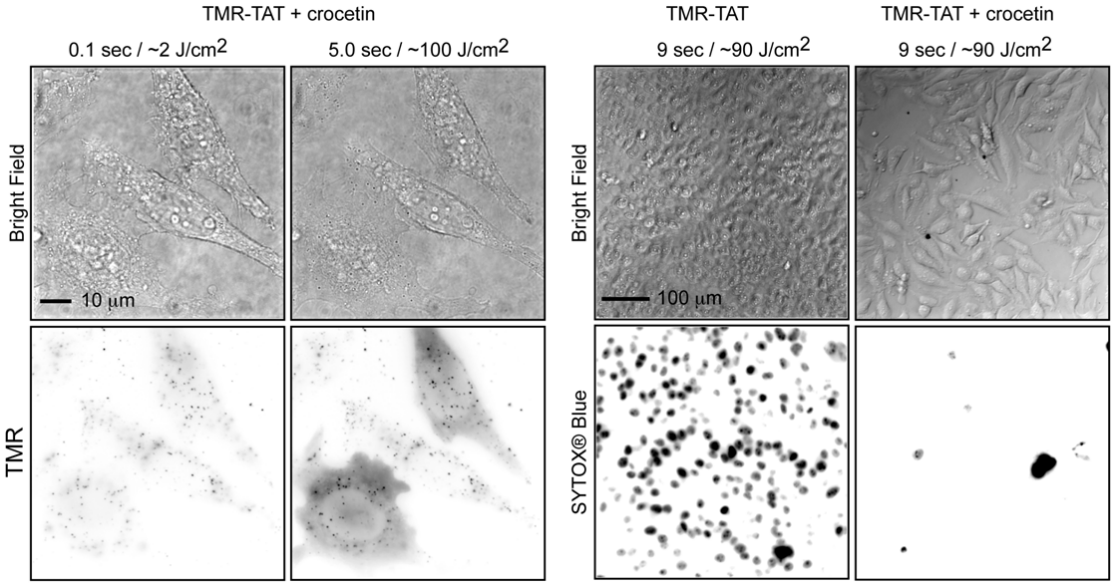
Crocetin inhibits photosensitization of TMR-TAT. TMR-TAT (3 µM) was incubated with crocetin (50 µM) and HeLa cells for 1 hour. Cells were washed with fresh L-15, placed on the microscope, and irradiated at 560 nm for the times indicated. The left panel represents cells imaged with the 100× and illustrates that endosomal release of TMR-TAT appears reduced by crocetin but not abolished. However, endosomal release is not accompanied by membrane blebbing or SYTOX® Blue staining (no signal detected, not shown) as seen when crocetin is omitted (right panel, cells are imaged with 20× objective).

To determine whether oxygen is involved in the light-induced lysis of RBCs mediated by TMR-TAT, oxygen was removed from the cell culture by degassing the incubation media with nitrogen. Under these conditions, the photohemolysis of RBCs exposed to TMR-TAT was greatly reduced (Fig. 5B). Co-incubation of TMR-TAT or TMR-riTAT (3 µM) with up to 50 mM sodium azide or L-histidine, two hydrophilic ^1^O_2_ scavengers, caused no detectable decrease in photohemolysis [17]. In contrast, addition of crocetin (50 µM), an amphiphilic carotenoid that inhibits ^1^O_2_ formation, led to a dramatic reduction in photohemolysis (Fig. 5B) [18]. A similar effect was observed upon addition of α-tocopherol acetate (60 µM), a lipophilic ^1^O_2_ quencher known to accumulate in membranes and to inhibit lipid peroxidation [19], [20]. A possible explanation for these observations might therefore be that crocetin and α-tocopherol acetate are able to quench singlet oxygen present in the hydrophobic environment of the membrane while other quenchers are not (Fig. 5B). In order to test whether ^1^O_2_ is also involved in the phototoxicity observed within HeLa cells, cells were incubated with crocetin or α-tocopherol acetate prior to incubation with TMR-TAT and during light irradiation. In the case of α-tocopherol acetate, photo-induced endosomal release and cell death were still observed (data not shown). The photosensitization of TMR-TAT was, however, delayed. This is consistent with this quencher being consumed during the experiment because of its reaction with ^1^O_2_. In contrast, crocetin inhibits ^1^O_2_ formation without being degraded. When using crocetin, endosomal release of the peptide could be observed, although at a lesser extent than when crocetin is not present (e.g. many fluorescent endosomes remain present during the experiment, Fig. 6). However, cell-death was not observed under these conditions; cells did not display membrane blebbing and did not become permeable to SYTOX® Blue (Fig. 6). In addition, photobleaching of TMR-TAT was observed when light exposure was increased. It is difficult to determine why crocetin did not inhibit endosomal release. On the other hand, this experiment suggests that the lysis of endocytic organelles is not sufficient to cause cell death. Overall, these results suggest that singlet oxygen is involved in the membrane disruption and cell death induced by light irradiation of TMR-TAT. They also suggest that the singlet oxygen responsible for membrane damage is generated directly in the lipid bilayer, since hydrophilic quenchers have no effects.

## Discussion

Upon light irradiation TMR-TAT rapidly and efficiently disrupts the membrane of endosomes. However, in our hands, this activity is also accompanied by the simultaneous destruction of cells. The peptide therefore played the role of a photosensitizer, a molecule able to kill cells upon light irradiation. While multiple cellular events might take place during the photosensitization of the compound, the damage to cellular membranes other than that of endosomes appears to lead to this photocytotoxic effect. The peptide was for instance able to lyse the plasma membrane of HeLa cells as well as the membrane of red blood cells upon light irradiation. The photocytotoxicity of the peptide also involves the formation of singlet oxygen upon light irradiation. Singlet oxygen is short-lived (lifetime <4 µs) and diffuses across only very short distances (singlet oxygen would travel a distance of ∼220 nm in water during three lifetimes and presumably much less when reacting with biomolecules inside a cell) [21]. To our knowledge, the singlet oxygen quantum yield of TMR has not been determined. However, triplet quantum yields ranging from 0.001 to 0.003 have been reported [22], [23]. Because formation of the fluorophore's triplet state is required for ^1^O_2_ formation, the singlet oxygen quantum yield of TMR should therefore be very low. Consequently, TMR would not be expected to act as an efficient photosensitizer. Indeed, TMR and TMR-K9 were essentially non-cytotoxic to cells under the conditions tested. Yet, TMR-TAT can reproduce some of the photo-induced effects obtained with hematoporphyrin, a prototypical photosensitizer with a singlet oxygen quantum yield of approximately 0.5 [11], [24]. TAT, therefore, appears to greatly enhance the phototoxicity of TMR. One possible explanation for this phenomenon might be related to how TAT interacts with membranes. TAT is known to bind heparan sulfate proteoglycans on the surface of cells and interactions with these species are important for the endocytosis of the peptide [25], [26], [27], [28]. The surface of RBCs is, however, depleted in heparan sulfate proteoglycans [29], [30]. The photolysis achieved with RBCs would therefore suggest that binding to heparan sulfate proteoglycans is not required for efficient photosensitization and that other membrane interactions are involved. Interestingly, TAT has also been shown to bind to the negatively charged head groups of phospholipids and to float on lipid bilayers at the water/lipid interface [31]. While the peptide does not appear to be strongly associated with cellular membranes in our fluorescence assays, it is, however, possible that a fraction of TMR-TAT resides close to or at the lipid bilayer as indicated by the crenation of erythrocytes. TAT might then position TMR in a membrane environment that the soluble TMR or TMR-K9 do not access. Interestingly, the membrane affinity of lipophilic porphyrin-based photosensitizers is thought to be an important feature of their photocytotoxicity [11], [32]. It has been proposed, for instance, that singlet oxygen generated in membranes reacts with membrane proteins or unsaturated lipids to form hydroperoxides according to the ene reaction [21]. This damage to membrane constituents is sufficient to cause membrane lysis and cell death [33], [34]. In addition, degradation of the formed lipid hydroperoxides might initiate the auto-catalyzed oxidation of neighboring lipids and cause a propagation of the damage done to the lipid bilayer through a chain reaction [19], [35]. A similar phenomenon might take place for TMR-TAT. While singlet oxygen might be produced in low yield upon irradiation, it might nonetheless be produced in a membrane environment more susceptible to damage. Our experiments suggest that singlet oxygen might be generated in a hydrophobic environment. The amphiphilic crocetin and the hydrophobic α-tocopherol acetate inhibits the photocytotoxicity of TMR-TAT, but soluble singlet oxygen quenchers such sodium azide, L-histidine, and glutathione do not. The membrane blebbing of HeLa cells and the shrinkage of RBCs further support the notion that the cell membranes and their constituents are being damaged. It is clear that photosensitization of TMR initiates these effects. Yet it is possible that TAT, beyond targeting TMR to critical cellular environments, might also play a direct role in the destruction of membranes. TAT is, for instance, able to cross lipid bilayers and, while the exact mechanisms involved in this process remain unclear, this activity is thought to involve the formation of transient pores in lipid bilayers [36], [37]. TAT could therefore aggravate the initial damage done by singlet oxygen. Elucidating whether this is the case will be the object of further studies.

The peptide had many desirable properties: photo-induced endosomal release required only short illumination with light of moderate intensities (as opposed to long irradiation time with intense lasers), this phenomenon happened in all irradiated cells, and a large fraction of the material endocytosed was released into the cytosolic space. On the other hand, in our assays, photo-induced endosomal release could not be achieved without cell-death occurring either simultaneously or with a short delay. The photocytoxicity associated with TMR-TAT constitutes a significant problem when one considers using this peptide in the context of photo-induced delivery of macromolecules into live cells. Addition of crocetin to the media might help solve this problem as crocetin inhibited the photocytotoxicity of TMR-TAT. However, the efficiency of endosomal release was also reduced by this treatment. In order to maximize the potential of this delivery approach, a future challenge will be to identify efficient photo-endosomolytic peptides that do not cause cell-death. Future investigations into the exact mechanisms by which TMR-TAT functions should help in the rational design of such compounds.

Despite being a problem for delivery applications, the photocytotoxicity of TMR-TAT might have interesting applications in Photodynamic therapy [38]. Photodynamic therapy (PDT) is a form of treatment that uses light to kill cells. PDT is used for the treatment of various cancers as well as for the management of diseases such as acne, macular degeneration, and arthritis [39], [40], [41], [42]. In a typical PDT protocol, a patient is treated with a photosensitizer, and tissues are selectively destroyed by restricting light irradiation to a local area. Interestingly, TAT and arginine-rich peptides have been conjugated to PDT photosensitizers to improve the delivery of these compounds to mammalian or bacterial cells [43], [44], [45]. A common assumption in these reports, and in this field in general, is that a high singlet oxygen quantum yield is a required characteristic for an ideal PDT photosensitizer. This is based on the idea that the more singlet oxygen is generated, the more cellular damage is achieved. Our results suggest, however, that addition of TAT to a chromophore can compensate for a low singlet oxygen quantum yield and convert the innocuous TMR compound into a potent photosensitizer. Overall, this means that TAT can, in addition to improving the cellular distribution of known PDT photosensitizers, increase the photodynamic potential of compounds that would otherwise not be considered as photosensitizers. This principle might lead to significant opportunities for the development of novel peptide-based PDT agents.

## Supporting Information

Figure S1 Toxicity of TMR-TAT toward HeLa cells in the absence of light. TMR-TAT was incubated at the concentration indicated with cells for 1 hour in L-15 at 37°C. Cells were washed with fresh L-15 and incubated for an additional 4 h. Cells were then treated with L-15 containing DAPI and SYTOX® green. DAPI stains the nucleus of all cells while SYTOX® green only stains the nucleus of dead cells. Cells were imaged by fluorescence microscopy using the DAPI and FITC filters to detect DAPI and SYTOX® green, respectively. For each experiments, five representative images were acquired using the 20× objective and the percentage of dead cells were calculated from the ratio of cells stained by SYTOX® green divided by the number of cells stained by DAPI. The reported data is the average of 3 experiments (3×5 images, ≥1000 cells/experiments) and the error bar represents the standard deviation.
(TIF)

Figure S2 Photosensitization of TMR-riTAT endocytosed by HeLa cells. HeLa cells were incubated with TMR-riTAT (3 µM) for 1 h and washed with fresh L-15 media. Cells were then incubated with L-15 containing 1 µM SYTOX® Blue to detect cells with compromised plasma membranes. Cells were observed using a 100× objective using bright field and fluorescence imaging (RFP filter to detect TMR-riTAT, pseudocolored red, and CFP filter to detect SYTOX® Blue, pseudo colored blue). The images are the overlay of TMR and bright field images and the insert images are the overlay of SYTOX® Blue and bright field images. At a low exposure dose, TMR-riTAT is distributed in a punctate manner within cells and cells are impermeable to SYTOX® Blue. As with TMR-TAT, TMR-riTAT is however quickly redistributed thought the cell as light exposure is increased. As this take place, the cell shrinks and membrane blebs form. The nuclei of cells are also stained by SYTOX® Blue, indicating that the plasma membrane integrity is compromised.
(TIF)

Figure S3 Comparison of the fluorescence intensity of endosomes containing TMR-TAT (incubation at 3 µM) or TMR-K9 (incubation at 10 µM) in the images presented in Figure 1. Imaging was performed in both cases under identical conditions. The fluorescence intensities of all endocytic organelles was measured the Slidebook software. These data show that the amount of TMR-K9 present in endocytic organelles is typically equal or greater that that of TMR-TAT (TMR-K9 however requires a greater concentration in the incubation media to achieve this result). These results therefore validate that the reduced activity seen with TMR-K9 when compared to TMR-TAT is not due to the fact that less material is present inside endocytic organelles.
(TIF)

Figure S4 Light irradiation alone does not cause cell-death. TMR-TAT induces cell-death upon light irradiation in HeLa or COS-7 cells. A) The conditions of light irradiation used in Figure 2A do not affect cell viability. HeLa cells were prepared as in Figure 2A except that TMR-TAT was omitted during the 1 h incubation in L-15. Cells were observed using a 10× objective using bright field and fluorescence imaging (CFP filter to detect SYTOX® Blue). The cells within the circled area were exposed to light at 560 nm for 10 sec (10× the exposure time used in Figure 2A). Cells were then imaged 5 min after irradiation to allow for SYTOX® Blue staining. Nuclei stained by SYTOX® Blue are represented as black dots in the inverted monochrome image. In contrast to Figure 2A, the cells within the irradiated area do not become stained with SYTOX® Blue after exposure to light. B) Similar experiment as in A) but with cells incubated with TMR-TAT. C) Photosensitization of TMR-TAT endocytosed by COS-7 cells. Experimental conditions were identical to those of Figure 2B. Cells were observed using a 100× objective using bright field and fluorescence imaging (RFP filter to detect TMR-TAT, pseudocolored red, and CFP filter to detect SYTOX® Blue, pseudo colored blue). As with HeLa cells, TMR-TAT is distributed in a punctate manner within cells at a low exposure dose and cells are impermeable to SYTOX® Blue. TMR-TAT is however quickly redistributed thought the cell as light exposure is increased. After 1 second of irradiation, membrane blebs are formed on the cell surface, and the nuclei of cells are stained by SYTOX® Blue.
(TIF)

Figure S5 A) Photohemolysis of RBCs with TMR-TAT. Short light exposure tat 560 nm to RBCs incubated with TMR-TAT (3 µM) causes lysis and formation of ghost cells as observed by bright field imaging. Lysis of more than 90% of the cells can be achieved when light exposure is increased. Initially, the ghosts formed appear to have a constant diameter as shown in the images corresponding to exposure at 20 J/cm^2^ and 60 J/cm^2^. As light exposure is increased, the ghosts shrink to a much smaller diameter (120 J/cm^2^ image). This shrinkage was not observed when the ghosts formed by irradiation with 60 J/cm^2^ of light were incubated without additional light irradiation (data not shown). These results suggest that light irradiation causes damages to membranes well after lysis as occurred. B) TMR-TAT does not appear to penetrate RBCs. RBCs were incubated with TMR-TAT (10 µM) in PBS for 1 hour. The RBCs were then spun down at low speed and the supernatant was removed from the pelleted cells. Cells were rapidly washed with cold PBS (4°C) and spun down twice. Images are the bright field and TMR confocal fluorescence images before and after washing of the RBCs. During incubation, the interior of RBCs display a dark contrast when compared to the fluorescent peptide present in solution. After washing the cells, no appreciable TMR fluorescence could be detected. It is important to note that RBCs have a weak autofluorescence signal in the TMR channel. The contrast in the image represented was therefore adjusted to display a signal that would be above this autofluorescence background. In addition, light irradiation of the washed cells did not lead to photohemolysis. Together, these results suggest that TMR-TAT does not penetrate RBCs to a large extent.
(TIF)

## References

[1] Zorko M, Langel U (2005). Cell-penetrating peptides: mechanism and kinetics of cargo delivery.. Adv Drug Deliv Rev.

[2] Maiolo JR, Ottinger EA, Ferrer M (2004). Specific redistribution of cell-penetrating peptides from endosomes to the cytoplasm and nucleus upon laser illumination.. J Am Chem Soc.

[3] Matsushita M, Noguchi H, Lu YF, Tomizawa K, Michiue H (2004). Photo-acceleration of protein release from endosome in the protein transduction system.. FEBS Lett.

[4] Endoh T, Sisido M, Ohtsuki T (2009). Spatial regulation of specific gene expression through photoactivation of RNAi.. J Control Release.

[5] Woods LK, Morgan RT, Quinn LA, Moore GE, Semple TU (1979). Comparison of four new cell lines from patients with adenocarcinoma of the ovary.. Cancer Res.

[6] Duchardt F, Fotin-Mleczek M, Schwarz H, Fischer R, Brock R (2007). A comprehensive model for the cellular uptake of cationic cell-penetrating peptides.. Traffic.

[7] Ruben S, Perkins A, Purcell R, Joung K, Sia R (1989). Structural and functional characterization of human immunodeficiency virus tat protein.. J Virol.

[8] Allison AC, Magnus IA, Young MR (1966). Role of lysosomes and of cell membranes in photosensitization.. Nature.

[9] Wilson PD, Firestone RA, Lenard J (1987). The role of lysosomal enzymes in killing of mammalian cells by the lysosomotropic detergent N-dodecylimidazole.. J Cell Biol.

[10] Berg K, Moan J (1994). Lysosomes as photochemical targets.. Int J Cancer.

[11] Valenzeno DP (1987). Photomodification of Biological-Membranes with Emphasis on Singlet Oxygen Mechanisms.. Photochemistry and Photobiology.

[12] Colin FC, Schrier SL (1991). Spontaneous endocytosis in human neonatal and adult red blood cells: comparison to drug-induced endocytosis and to receptor-mediated endocytosis.. Am J Hematol.

[13] Birchmeier W, Lanz JH, Winterhalter KH, Conrad MJ (1979). ATP-induced endocytosis in human erythrocyte ghosts. Characterization of the process and isolation of the endocytosed vesicles.. J Biol Chem.

[14] Jay AW, Rowlands S (1975). The stages of osmotic haemolysis.. J Physiol.

[15] Sheetz MP, Singer SJ (1974). Biological membranes as bilayer couples. A molecular mechanism of drug-erythrocyte interactions.. Proc Natl Acad Sci U S A.

[16] Evans EA (1974). Bending resistance and chemically induced moments in membrane bilayers.. Biophys J.

[17] Davies MJ (2004). Reactive species formed on proteins exposed to singlet oxygen.. Photochem Photobiol Sci.

[18] Reyftmann JP, Kohen E, Morliere P, Santus R, Kohen C (1986). A microspectrofluorometric study of porphyrin-photosensitized single living cells–I. Membrane alterations.. Photochem Photobiol.

[19] Morliere P, Moysan A, Santus R, Huppe G, Maziere JC (1991). UVA-induced lipid peroxidation in cultured human fibroblasts.. Biochim Biophys Acta.

[20] Ouedraogo GD, Redmond RW (2003). Secondary reactive oxygen species extend the range of photosensitization effects in cells: DNA damage produced via initial membrane photosensitization.. Photochem Photobiol.

[21] Redmond RW, Kochevar IE (2006). Spatially resolved cellular responses to singlet oxygen.. Photochem Photobiol.

[22] Eggeling C, Widengren J, Rigler R, Seidel CAM (1998). Photobleaching of fluorescent dyes under conditions used for single-molecule detection: Evidence of two-step photolysis.. Analytical Chemistry.

[23] Geissbuehler M, Spielmann T, Formey A, Marki I, Leutenegger M (2010). Triplet Imaging of Oxygen Consumption during the Contraction of a Single Smooth Muscle Cell (A7r5).. Biophysical Journal.

[24] Blum A, Grossweiner LI (1985). Singlet Oxygen Generation by Hematoporphyrin-Ix, Uroporphyrin-I and Hematoporphyrin Derivative at 546 Nm in Phosphate Buffer and in the Presence of Egg Phosphatidylcholine Liposomes.. Photochemistry and Photobiology.

[25] Nakase I, Tadokoro A, Kawabata N, Takeuchi T, Katoh H (2007). Interaction of arginine-rich peptides with membrane-associated proteoglycans is crucial for induction of actin organization and macropinocytosis.. Biochemistry.

[26] Tyagi M, Rusnati M, Presta M, Giacca M (2001). Internalization of HIV-1 tat requires cell surface heparan sulfate proteoglycans.. J Biol Chem.

[27] Console S, Marty C, Garcia-Echeverria C, Schwendener R, Ballmer-Hofer K (2003). Antennapedia and HIV transactivator of transcription (TAT) “protein transduction domains” promote endocytosis of high molecular weight cargo upon binding to cell surface glycosaminoglycans.. J Biol Chem.

[28] Poon GM, Gariepy J (2007). Cell-surface proteoglycans as molecular portals for cationic peptide and polymer entry into cells.. Biochem Soc Trans.

[29] Drzeniek Z, Stocker G, Siebertz B, Just U, Schroeder T (1999). Heparan sulfate proteoglycan expression is induced during early erythroid differentiation of multipotent hematopoietic stem cells.. Blood.

[30] Vogt AM, Winter G, Wahlgren M, Spillmann D (2004). Heparan sulphate identified on human erythrocytes: a Plasmodium falciparum receptor.. Biochem J.

[31] Ciobanasu C, Harms E, Tunnemann G, Cardoso MC, Kubitscheck U (2009). Cell-penetrating HIV1 TAT peptides float on model lipid bilayers.. Biochemistry.

[32] Grossweiner LI (1984). Membrane photosensitization by hematoporphyrin and hematoporphyrin derivative.. Prog Clin Biol Res.

[33] Eisenberg WC, Taylor K, Grossweiner LI (1984). Lysis of egg phosphatidylcholine liposomes by singlet oxygen generated in the gas phase.. Photochem Photobiol.

[34] Doleiden FH, Fahrenholtz SR, Lamola AA, Trozzolo AM (1974). Reactivity of cholesterol and some fatty acids toward singlet oxygen.. Photochem Photobiol.

[35] Sevanian A, Hochstein P (1985). Mechanisms and consequences of lipid peroxidation in biological systems.. Annu Rev Nutr.

[36] Herce HD, Garcia AE (2007). Molecular dynamics simulations suggest a mechanism for translocation of the HIV-1 TAT peptide across lipid membranes.. Proc Natl Acad Sci U S A.

[37] Herce HD, Garcia AE, Litt J, Kane RS, Martin P (2009). Arginine-rich peptides destabilize the plasma membrane, consistent with a pore formation translocation mechanism of cell-penetrating peptides.. Biophys J.

[38] Dolmans DE, Fukumura D, Jain RK (2003). Photodynamic therapy for cancer.. Nat Rev Cancer.

[39] Brown SB, Brown EA, Walker I (2004). The present and future role of photodynamic therapy in cancer treatment.. Lancet Oncol.

[40] Hong SB, Lee MH (2005). Topical aminolevulinic acid-photodynamic therapy for the treatment of acne vulgaris.. Photodermatol Photoimmunol Photomed.

[41] Schmidt-Erfurth U, Miller JW, Sickenberg M, Laqua H, Barbazetto I (1999). Photodynamic therapy with verteporfin for choroidal neovascularization caused by age-related macular degeneration: results of retreatments in a phase 1 and 2 study.. Arch Ophthalmol.

[42] Bagdonas S, Kirdaite G, Streckyte G, Graziene V, Leonaviciene L (2005). Spectroscopic study of ALA-induced endogenous porphyrins in arthritic knee tissues: targeting rheumatoid arthritis PDT.. Photochem Photobiol Sci.

[43] Bourre L, Giuntini F, Eggleston IM, Mosse CA, Macrobert AJ (2010). Effective photoinactivation of Gram-positive and Gram-negative bacterial strains using an HIV-1 Tat peptide-porphyrin conjugate.. Photochem Photobiol Sci.

[44] Choi Y, McCarthy JR, Weissleder R, Tung CH (2006). Conjugation of a photosensitizer to an oligoarginine-based cell-penetrating peptide increases the efficacy of photodynamic therapy.. Chem Med Chem.

[45] Sehgal I, Sibrian-Vazquez M, Vicente MG (2008). Photoinduced cytotoxicity and biodistribution of prostate cancer cell-targeted porphyrins.. J Med Chem.

