# Supplementary figures and images for: Conjugation to the Cell-Penetrating Peptide TAT Potentiates the Photodynamic Effect of Carboxytetramethylrhodamine
Source: PLoS One. 2011 Mar 14;6(3):e17732. doi: 10.1371/journal.pone.0017732 (PMC3056768; doi:10.1371/journal.pone.0017732)

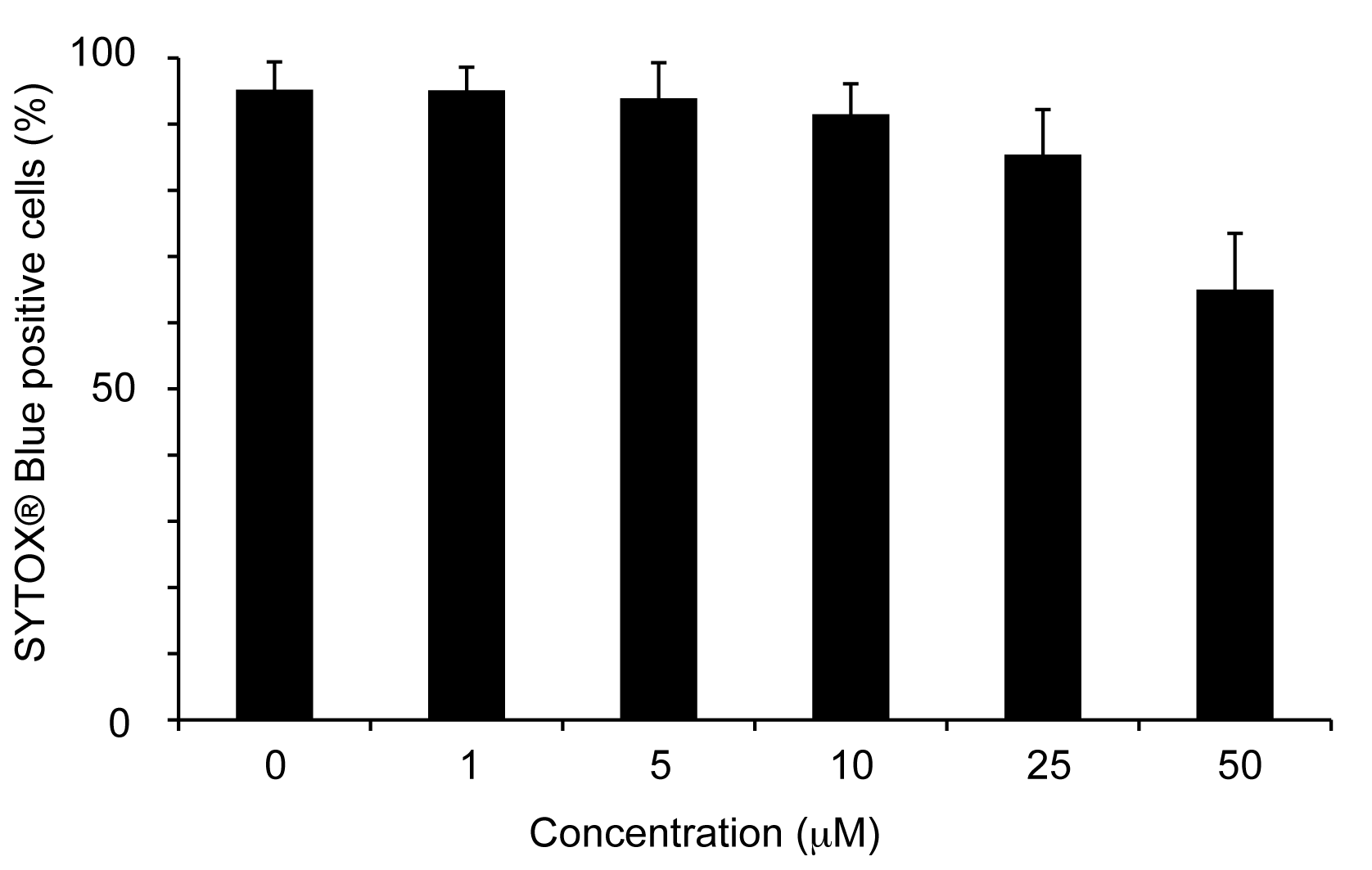

Supplement: Figure S1 — Toxicity of TMR-TAT toward HeLa cells in the absence of light. TMR-TAT was incubated at the concentration indicated with cells for 1 hour in L-15 at 37°C. Cells were washed with fresh L-15 and incubated for an additional 4 h. Cells were then treated with L-15 containing DAPI and SYTOX® green. DAPI stains the nucleus of all cells while SYTOX® green only stains the nucleus of dead cells. Cells were imaged by fluorescence microscopy using the DAPI and FITC filters to detect DAPI and SYTOX® green, respectively. For each experiments, five representative images were acquired using the 20× objective and the percentage of dead cells were calculated from the ratio of cells stained by SYTOX® green divided by the number of cells stained by DAPI. The reported data is the average of 3 experiments (3×5 images, ≥1000 cells/experiments) and the error bar represents the standard deviation. (TIF) [file pone.0017732.s001.tif]

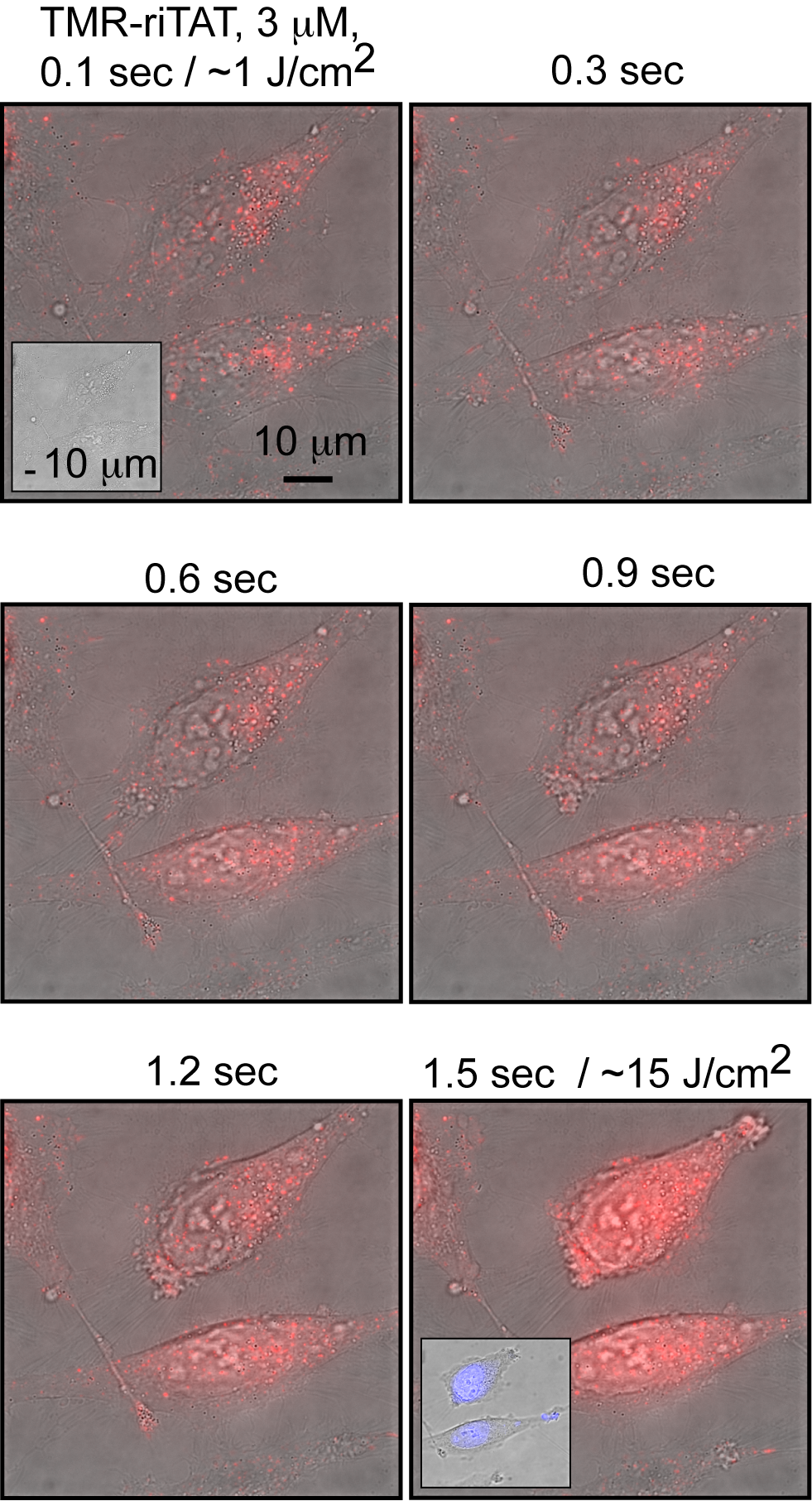

Supplement: Figure S2 — Photosensitization of TMR-riTAT endocytosed by HeLa cells. HeLa cells were incubated with TMR-riTAT (3 µM) for 1 h and washed with fresh L-15 media. Cells were then incubated with L-15 containing 1 µM SYTOX® Blue to detect cells with compromised plasma membranes. Cells were observed using a 100× objective using bright field and fluorescence imaging (RFP filter to detect TMR-riTAT, pseudocolored red, and CFP filter to detect SYTOX® Blue, pseudo colored blue). The images are the overlay of TMR and bright field images and the insert images are the overlay of SYTOX® Blue and bright field images. At a low exposure dose, TMR-riTAT is distributed in a punctate manner within cells and cells are impermeable to SYTOX® Blue. As with TMR-TAT, TMR-riTAT is however quickly redistributed thought the cell as light exposure is increased. As this take place, the cell shrinks and membrane blebs form. The nuclei of cells are also stained by SYTOX® Blue, indicating that the plasma membrane integrity is compromised. (TIF) [file pone.0017732.s002.tif]

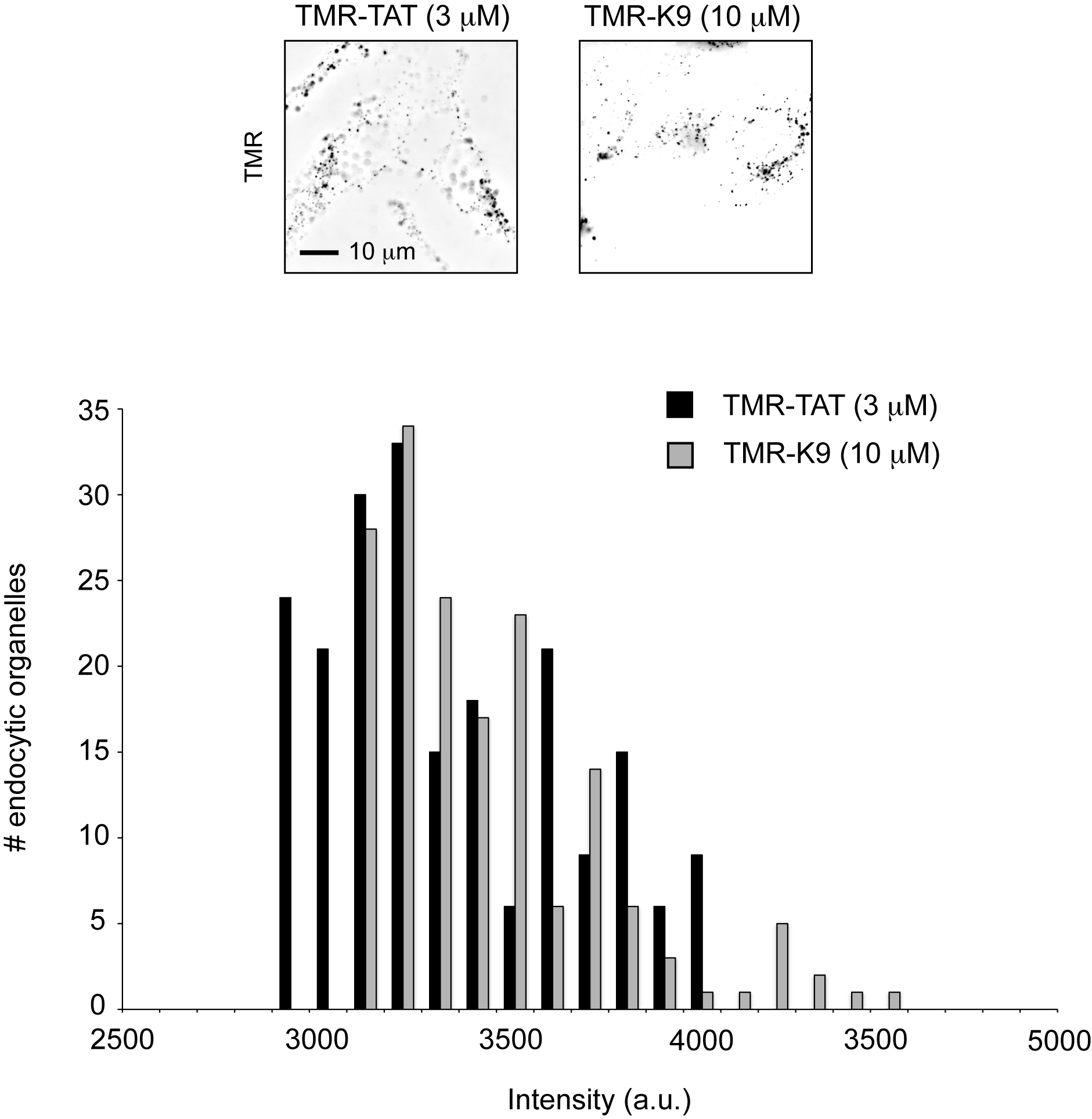

Supplement: Figure S3 — Comparison of the fluorescence intensity of endosomes containing TMR-TAT (incubation at 3 µM) or TMR-K9 (incubation at 10 µM) in the images presented in Figure 1. Imaging was performed in both cases under identical conditions. The fluorescence intensities of all endocytic organelles was measured the Slidebook software. These data show that the amount of TMR-K9 present in endocytic organelles is typically equal or greater that that of TMR-TAT (TMR-K9 however requires a greater concentration in the incubation media to achieve this result). These results therefore validate that the reduced activity seen with TMR-K9 when compared to TMR-TAT is not due to the fact that less material is present inside endocytic organelles. (TIF) [file pone.0017732.s003.tif]

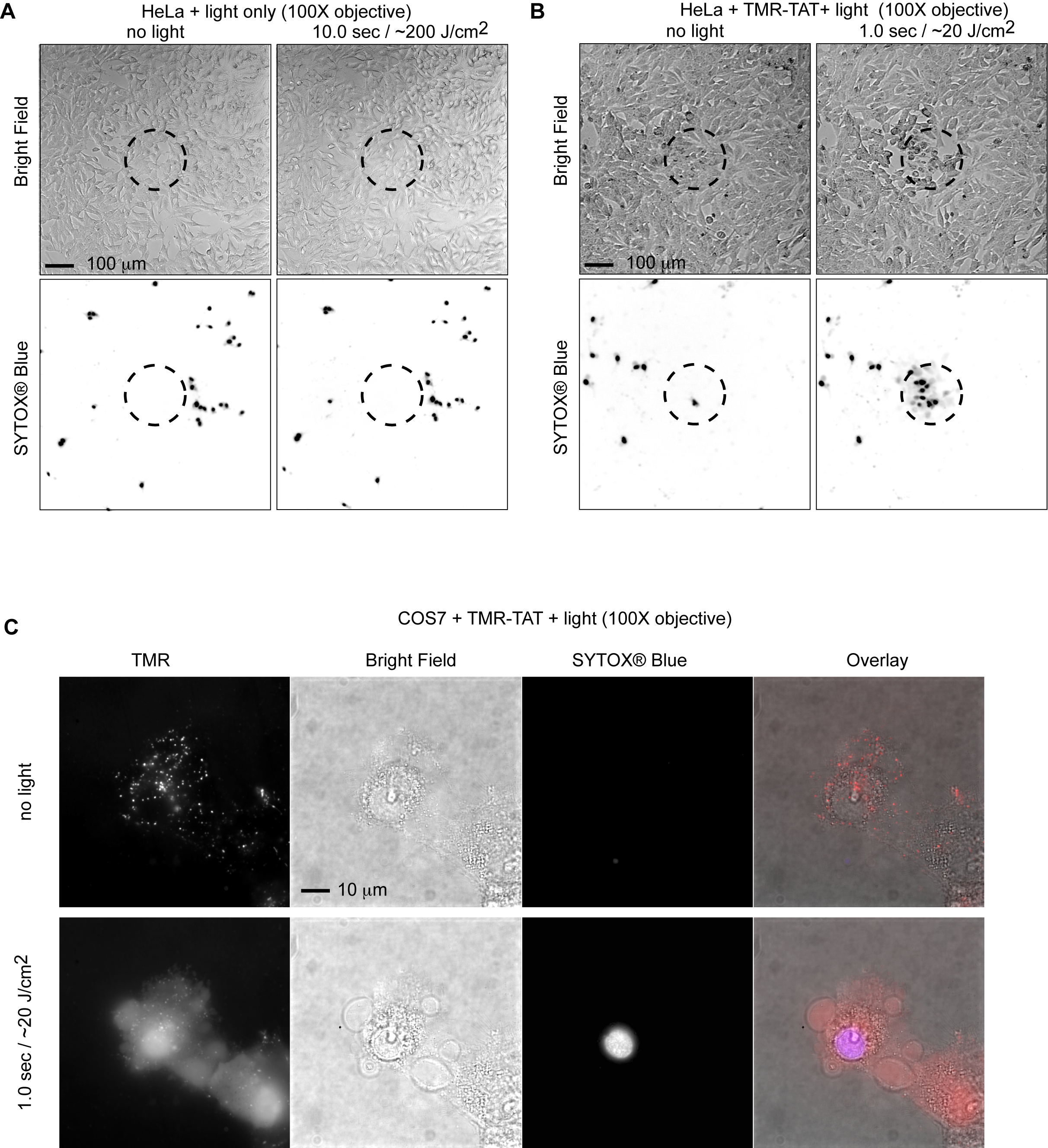

Supplement: Figure S4 — Light irradiation alone does not cause cell-death. TMR-TAT induces cell-death upon light irradiation in HeLa or COS-7 cells. A) The conditions of light irradiation used in Figure 2A do not affect cell viability. HeLa cells were prepared as in Figure 2A except that TMR-TAT was omitted during the 1 h incubation in L-15. Cells were observed using a 10× objective using bright field and fluorescence imaging (CFP filter to detect SYTOX® Blue). The cells within the circled area were exposed to light at 560 nm for 10 sec (10× the exposure time used in Figure 2A). Cells were then imaged 5 min after irradiation to allow for SYTOX® Blue staining. Nuclei stained by SYTOX® Blue are represented as black dots in the inverted monochrome image. In contrast to Figure 2A, the cells within the irradiated area do not become stained with SYTOX® Blue after exposure to light. B) Similar experiment as in A) but with cells incubated with TMR-TAT. C) Photosensitization of TMR-TAT endocytosed by COS-7 cells. Experimental conditions were identical to those of Figure 2B. Cells were observed using a 100× objective using bright field and fluorescence imaging (RFP filter to detect TMR-TAT, pseudocolored red, and CFP filter to detect SYTOX® Blue, pseudo colored blue). As with HeLa cells, TMR-TAT is distributed in a punctate manner within cells at a low exposure dose and cells are impermeable to SYTOX® Blue. TMR-TAT is however quickly redistributed thought the cell as light exposure is increased. After 1 second of irradiation, membrane blebs are formed on the cell surface, and the nuclei of cells are stained by SYTOX® Blue. (TIF) [file pone.0017732.s004.tif]

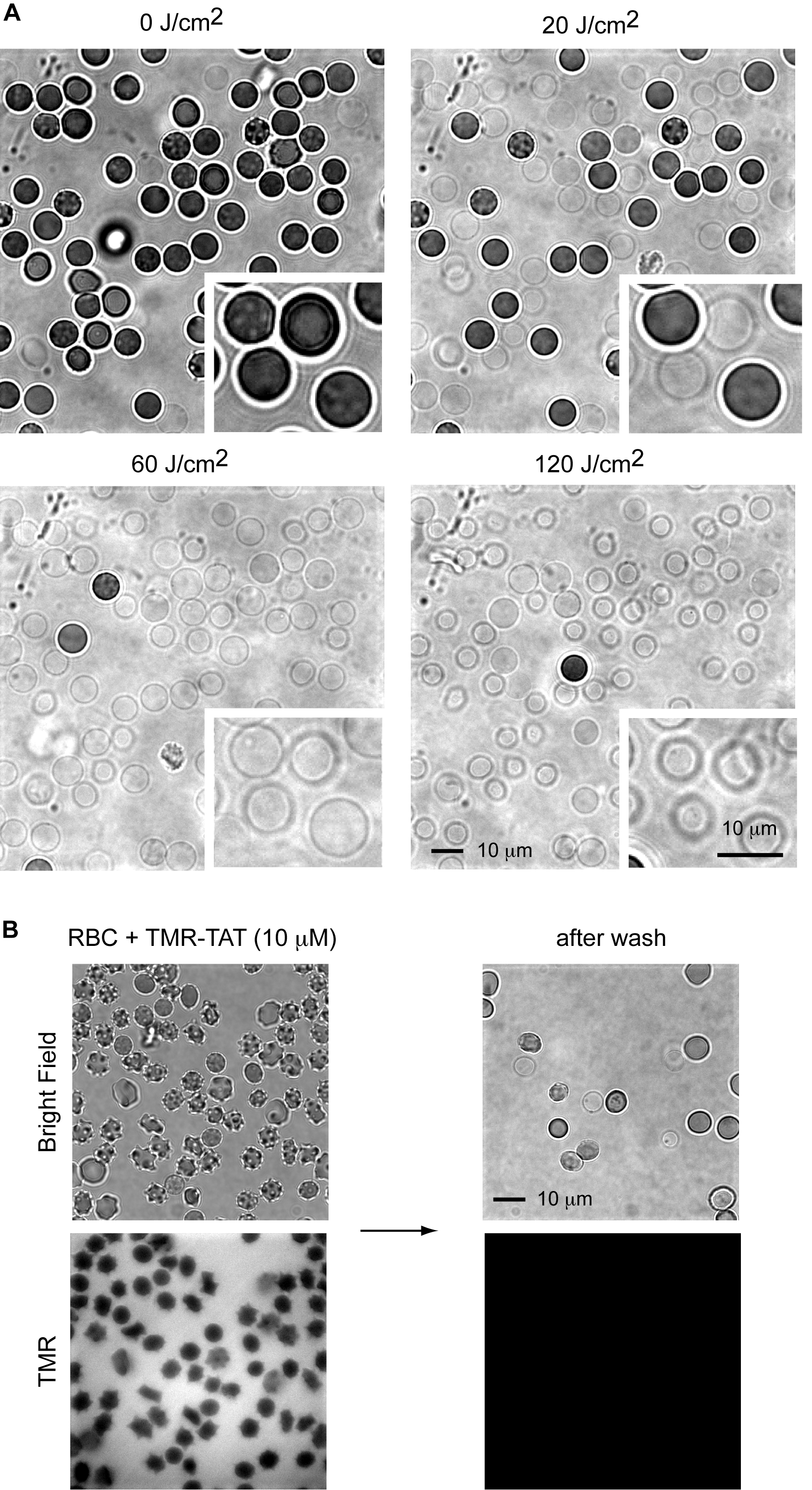

Supplement: Figure S5 — A) Photohemolysis of RBCs with TMR-TAT. Short light exposure tat 560 nm to RBCs incubated with TMR-TAT (3 µM) causes lysis and formation of ghost cells as observed by bright field imaging. Lysis of more than 90% of the cells can be achieved when light exposure is increased. Initially, the ghosts formed appear to have a constant diameter as shown in the images corresponding to exposure at 20 J/cm2 and 60 J/cm2. As light exposure is increased, the ghosts shrink to a much smaller diameter (120 J/cm2 image). This shrinkage was not observed when the ghosts formed by irradiation with 60 J/cm2 of light were incubated without additional light irradiation (data not shown). These results suggest that light irradiation causes damages to membranes well after lysis as occurred. B) TMR-TAT does not appear to penetrate RBCs. RBCs were incubated with TMR-TAT (10 µM) in PBS for 1 hour. The RBCs were then spun down at low speed and the supernatant was removed from the pelleted cells. Cells were rapidly washed with cold PBS (4°C) and spun down twice. Images are the bright field and TMR confocal fluorescence images before and after washing of the RBCs. During incubation, the interior of RBCs display a dark contrast when compared to the fluorescent peptide present in solution. After washing the cells, no appreciable TMR fluorescence could be detected. It is important to note that RBCs have a weak autofluorescence signal in the TMR channel. The contrast in the image represented was therefore adjusted to display a signal that would be above this autofluorescence background. In addition, light irradiation of the washed cells did not lead to photohemolysis. Together, these results suggest that TMR-TAT does not penetrate RBCs to a large extent. (TIF) [file pone.0017732.s005.tif]
